# Microscale spatial distributions of microbes and viruses in intertidal photosynthetic microbial mats

**DOI:** 10.1186/s40064-015-0977-8

**Published:** 2015-05-23

**Authors:** Cátia Carreira, Tim Piel, Marc Staal, Jan-Berend W Stuut, Mathias Middelboe, Corina P D Brussaard

**Affiliations:** Department of Biological Oceanography, Royal Netherlands Institute for Sea Research (NIOZ), PO Box 59, NL 1790 AB Den Burg, The Netherlands; Section for Marine Biology, University of Copenhagen, Strandpromenaden 5, 3000, Helsingør, Denmark; Department of Marine Geology and Chemical Oceanography, Royal Netherlands Institute for Sea Research (NIOZ), PO Box 59, NL 1790 AB Den Burg, The Netherlands; Department of Marine Geology, MARUM – Center for Marine Environmental Sciences, PO Box 330440, D 28334 Bremen, Germany; Aquatic Microbiology, Institute for Biodiversity and Ecosystem Dynamics, University of Amsterdam, Amsterdam, The Netherlands

**Keywords:** Viruses, Prokaryotes, Oxygenic photoautotrophs, Photosynthetic microbial mats, Spatial distribution

## Abstract

Intertidal photosynthetic microbial mats from the Wadden Sea island Schiermonnikoog were examined for microscale (millimetre) spatial distributions of viruses, prokaryotes and oxygenic photoautotrophs (filamentous cyanobacteria and benthic diatoms) at different times of the year. Abundances of viruses and prokaryotes were among the highest found in benthic systems (0.05–5.43 × 10^10^ viruses g^−1^ and 0.05–2.14 × 10^10^ prokaryotes g^−1^). The spatial distribution of viruses, prokaryotes and oxygenic photoautotrophs were highly heterogeneous at mm scales. The vertical distributions of both prokaryotic and viral abundances were related to the depth of the oxygenic photoautotrophic layer, implying that the photosynthetic mat fuelled the microbial processes in the underlying layer. Our data suggest that viruses could make an important component in these productive environments potentially affecting the biodiversity and nutrient cycling within the mat.

## Background

Microbial mats are laminated microbial communities growing in a variety of environments, including extreme habitats such as sea ice, hot springs and environments with high salinity or periodic desiccation (Castenholz 1994; Fenchel et al. 2012). Marine intertidal flats can sustain microbial mats under specific conditions, like occasional flooding and low sand deposition, (Bolhuis et al. 2015). Microbial mats are characterized by large chemical gradients and densely packed biomass within a few cm depth, growing an average of 1–3 mm per year, and covering sandy sediments in intertidal flats (Fenchel 1998; Fenchel and Kühl 2000; Bolhuis et al. 2015).

Filamentous cyanobacteria are considered the first colonisers in the development of intertidal photosynthetic microbial mats, forming a green layer that releases organic compounds (lysis, excretion) and oxygen, and captures atmospheric nitrogen (Stal 1995). Diatoms settle after, and together with the cyanobacteria make up the oxygenic photoautotrophic surface layer (Fenchel et al. 2012). Below this layer, bands of colourless sulfur and purple sulfur bacteria (purple layer) may develop, that oxidize sulfide generated by the underlying sulfate reducing bacteria (black layer) (Van Gemerden 1993). The intertwined filamentous cyanobacteria in the top layer and the excretion of exopolymeric substances (EPS) by the microbial communities form a matrix which stabilises the microbial mats, making them resistant to wind and wave erosion (De Brouwer et al. 2002). The development of mats is also enhanced by the reduced impact of grazers and bioturbating animals (Fenchel 1998) due to the relatively extreme physico-chemical conditions (e.g. hot springs, sea ice; dry and windy condition) inside and below the mat (Jørgensen et al. 1983; Des Marais 2003).

Studies in water column, biofilms, and sediments have demonstrated that microorganisms are heterogeneously distributed on small spatial scales with microscale hot spots of elevated activity (Neu and Lawrence 1997; Seymour et al. 2006; Stewart and Franklin 2008; Carreira et al. 2013). Moreover, studies have shown that to capture the actual microbial diversity, production, and the micro-environmental drivers of these habitats it is important to study microbial ecology at small scale (Paerl and Pinckney 1996; Azam and Malfatti 2007). In benthic environments, viruses were shown to play an important structuring role by driving spatial heterogeneity and temporal dynamics of their host populations (e.g. prokaryote; Siem-Jørgensen et al. 2008; Carreira et al. 2013), and as drivers of organic carbon and nutrient recycling (Danovaro et al. 2008b). Although photosynthetic microbial mats are highly productive habitats (Canfield et al. 2005) that have been intensively studied with regard to their structure, composition, and biogeochemistry (Des Marais 2003; Ward et al. 2006; Bolhuis et al. 2015), to the best of our knowledge, no studies on millimetre scale distribution of the different ecologically relevant groups in photosynthetic microbial mats have been published to date. The aim of the present study was to determine the microscale (mm) vertical and horizontal spatial distributions of viruses, prokaryotes (Archaea and Bacteria, excluding cyanobacteria) and oxygenic photoautotrophic (oxygenic photosynthetic microbes, i.e. prokaryotic filamentous cyanobacteria and eukaryotic microalgae) in intertidal photosynthetic microbial mats during different times of the year (November 2012, April and July 2013).

## Results

The intertidal zone on Schiermonnikoog was covered by photosynthetic microbial mats in an area of about 7 km^2^, and was characterised by periodical immersion during high tide. Typically, the sediment consisted of fairly homogeneous, well-sorted sands with a mean modal grain size between 180–220 μm. A 1 to 2 mm thick silty layer (modal size between 20–40 μm) was found at 4–5 mm depth. The water content of the sediment decreased strongly in the top 2 mm from 42 % in the top 0–1 mm, to 26 % in 1–2 mm depth layer, to 20 % at 2–10 mm deep. Organic matter content also decreased with depth, with 7 % at the top (0–1 mm), 2 % at the middle (1–5 mm) and 0.6 % at depth (5–10 mm).

**Fig. 1:**
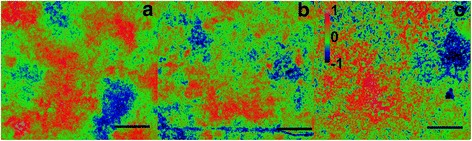
Autofluorescence of blue to amber ratio (BAR) images of the top view of the photosynthetic layer of the microbial mat on Schiermonnikoog (The Netherlands). **a** November, **b** April, **c** July. Colour scale indicates that values < 0 are cyanobacteria dominated and > 0 are diatom dominated. Scale bars are 1 cm

**Fig. 2:**
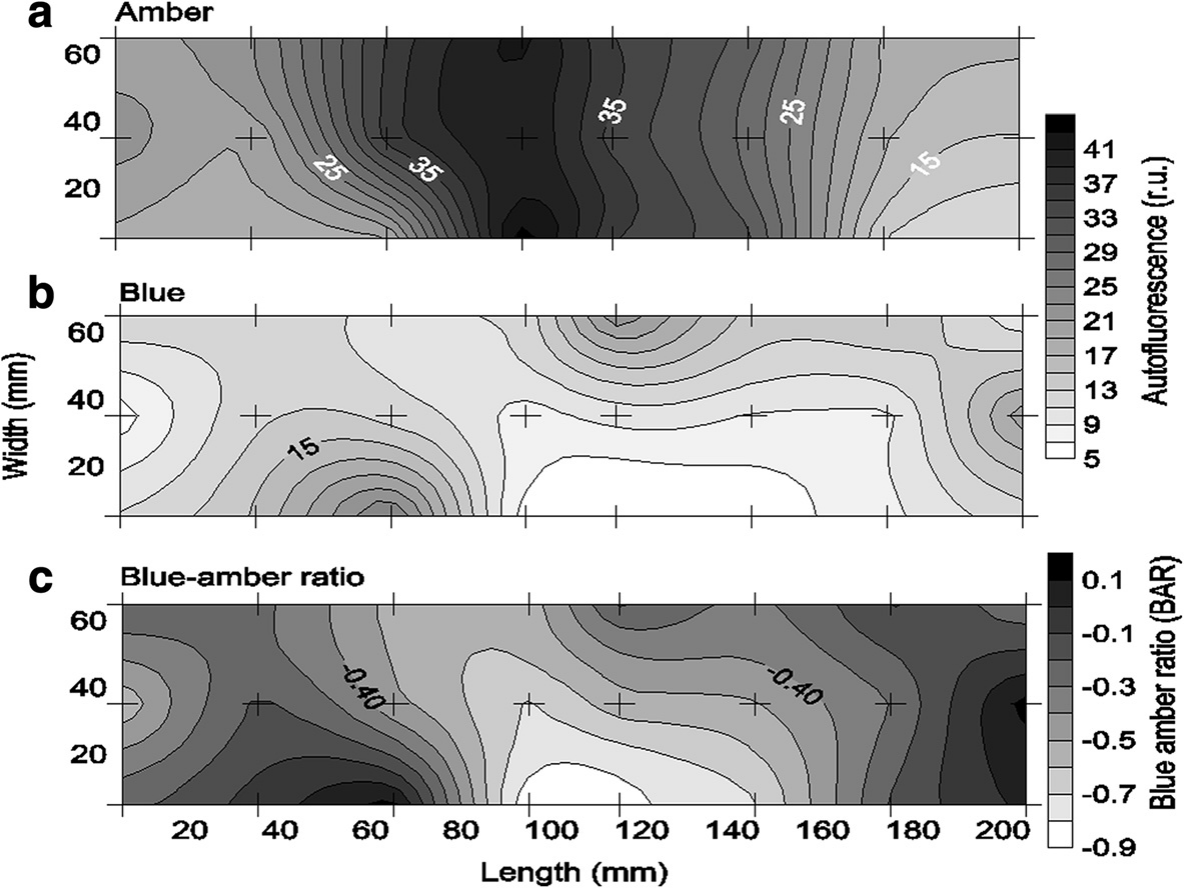
Spatial distribution of autofluorescence (relative units) distribution in the photosynthetic microbial mat on Schiermonnikoog in November 2012, after **a** amber (cyanobacteria) light exposure, **b** blue (diatoms) light exposure, and **c** blue to amber ratio (BAR). Area of studied sample: 210 × 70 × 1 mm (L × W × H). Crosses indicate sampling points

**Fig. 3:**
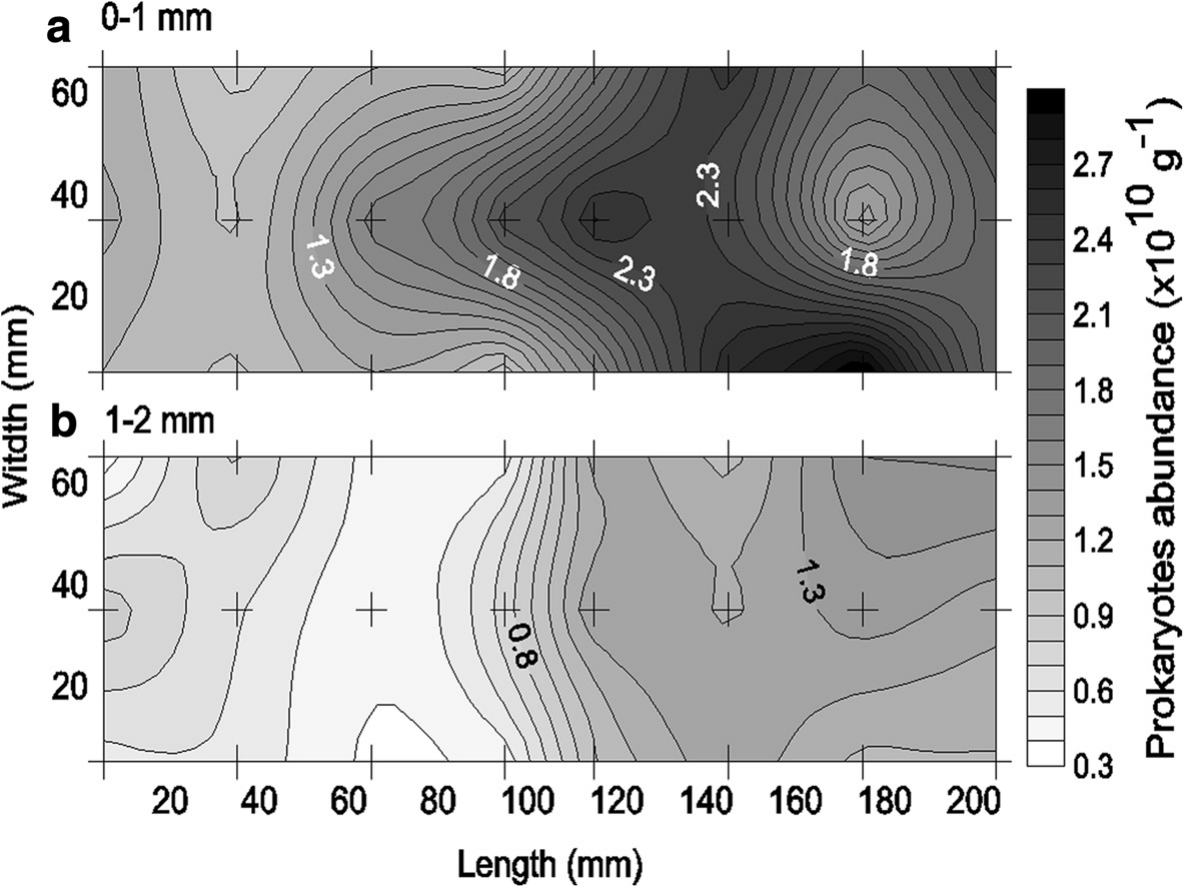
Spatial distribution of the prokaryotic abundance in **a** the top 0–1 mm, and **b** the lower 1–2 mm layer of a photosynthetic microbial mat on Schiermonnikoog (The Netherlands) in November 2012. Area of sampling was 210 × 70 × 2 mm (L × W × H). Crosses indicate the sampling points

**Fig. 4:**
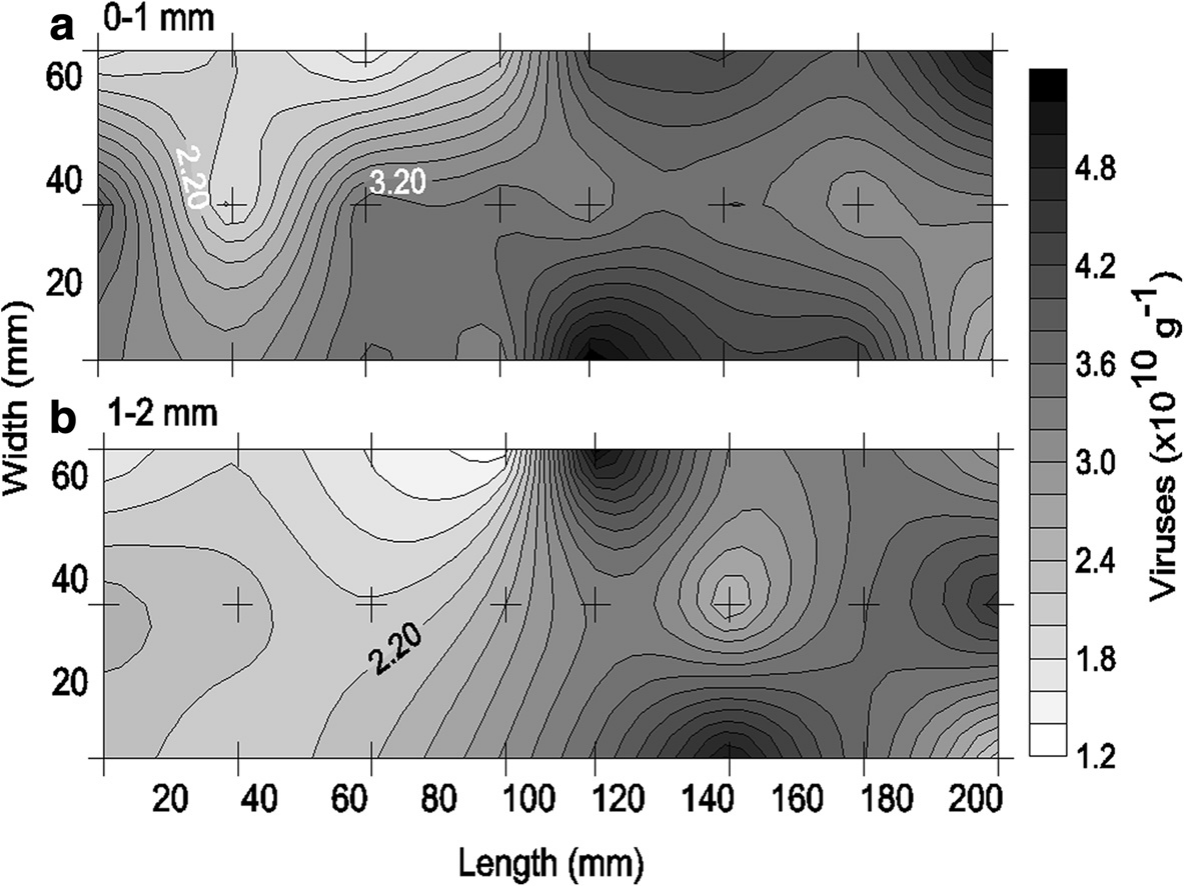
Spatial distribution of the viral abundance in **a** the top 0–1 mm, and **b** the lower 1–2 mm layer of a photosynthetic microbial mat on Schiermonnikoog (The Netherlands) in November 2012. Area of sampling was 210 × 70 × 2 mm (L × W × H). Crosses indicate the sampling points

**Fig. 5:**
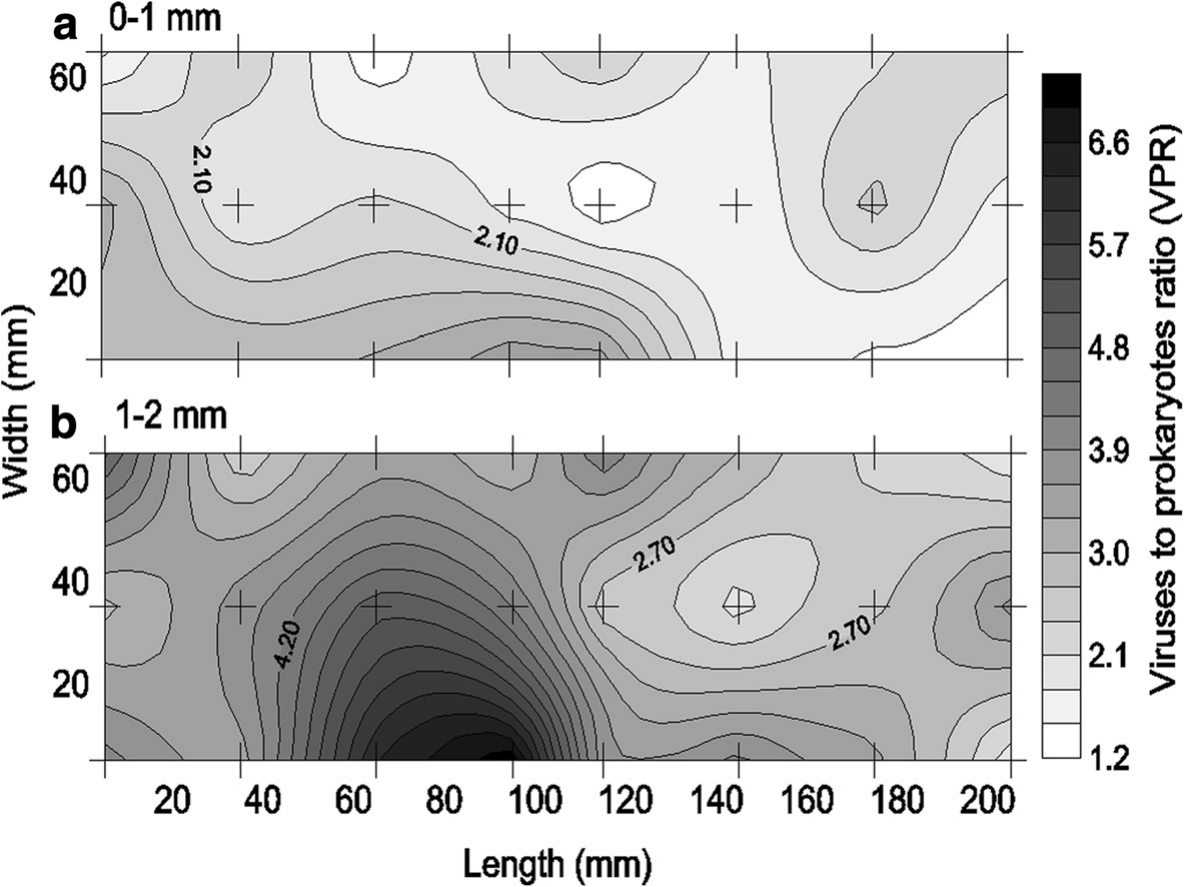
Spatial distribution of the viruses to prokaryotes ratio (VPR) in **a** the top 0–1 mm, and **b** the lower 1–2 mm layer of a photosynthetic microbial mat on Schiermonnikoog (The Netherlands) in November 2012. Area of sampling was 210 × 70 × 2 mm (L × W × H). Crosses indicate the sampling points

In general, the photosynthetic microbial mats were dominated by filamentous cyanobacteria and benthic diatoms as shown by BAR analysis (Fig. 1) and confirmed by light microscopy (green algae not observed). In the 3D study, cyanobacteria dominated the mat surface and were horizontally distributed in clusters separated by diatom clusters within the mm scale (Fig. 2). The average prokaryotic abundance (Fig. 3) in the November 3D-study was about 2-fold higher in the 0–1 mm top layer (1.7 ± 0.6 × 10^10^ g^−1^) compared to the 1–2 mm lower layer (0.9 ± 0.4 × 10^10^ g^−1^; p < 0.001). Viral abundances for the top 0–1 mm and the lower 1–2 mm, were however, rather similar, i.e. ranged from 1.4 to 5.4 × 10^10^ g^−1^ with averages of 3.3 ± 1.1 and 2.9 ± 1.1 × 10^10^ g^−1^, respectively (Fig. 4). Because prokaryotic abundance varied more (higher in top) than viral abundance, the average virus to prokaryotes ratio (VPR) was 1.6-fold lower (p < 0.001) in the top layer as compared to the bottom layer (3.5 ± 1.3), causing a VPR ‘hotspot’ (VPR = 7.2) in the lower layer (Fig. 5). Viral and prokaryotic abundances did not show significant correlation with oxygenic photoautotrophs (Fig. 4).

**Fig. 6:**
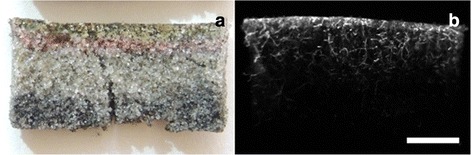
Cross section example of a typical photosynthetic mat from Schiermonnikoog (The Netherlands). **a** Colour image, **b** autofluorescence image after amber excitation showing the photosynthetic layer. Scale bar is 0.5 cm

**Fig. 7:**
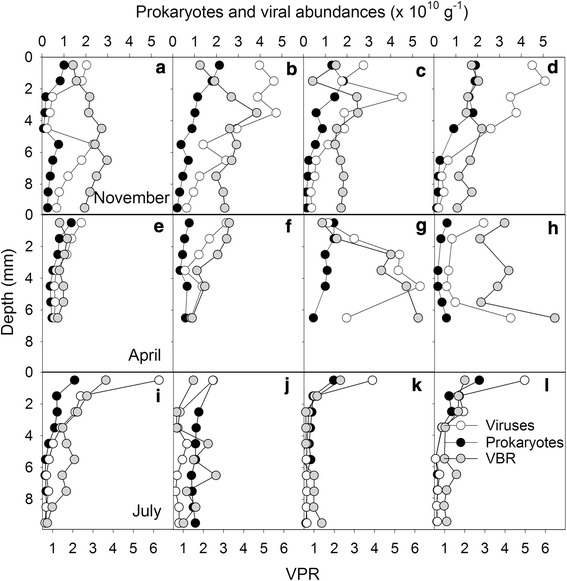
Depth profiles of the four replicates showing the abundances of viruses (open symbols) and prokaryotes (black symbols), and virus to prokaryotes ratio (VPR; grey symbols) in November (panel **a**, **b**, **c**, and **d**), April (panel **e**, **f**, **g**, and **h**) and July (panel **i**, **j**, **k**, and **l**) in Schiermonnikoog (The Netherlands)

The top photosynthetic layer decreased from 2 mm in November to 1 mm in April, and increased again in July to 1.5 mm (Fig. 6a, b). The anoxic black layer characterized by iron sulfide appeared 4–5 mm lower in April and July than at the 2 mm depth in November. Independent of season, a distinct purple sulfur bacteria layer of about 1 mm was occasionally observed below the oxygenic photoautotrophs. The vertical profiles in the abundances of prokaryotes and viruses varied with date of sampling (Fig. 7). Viral abundances were significantly different in November and April (p < 0.001) compared to July, whereas no differences were found between November and April viral abundances. Prokaryotes and VPR showed significantly different abundances in November compared to April (p < 0.05 and p < 0.001, respectively) and July (p < 0.001 and p < 0.05, respectively), but no differences were found between April and July. Overall, prokaryotes and viral abundances were highest in November, followed by April, and July. VPR was highest in November (average 2.7 ± 0.7, range 1.0–4.7), followed by April (1.8 ± 1.6; 0.5–5.4) and July (1.4 ± 0.7; 0.4–3.6).

**Fig. 8:**
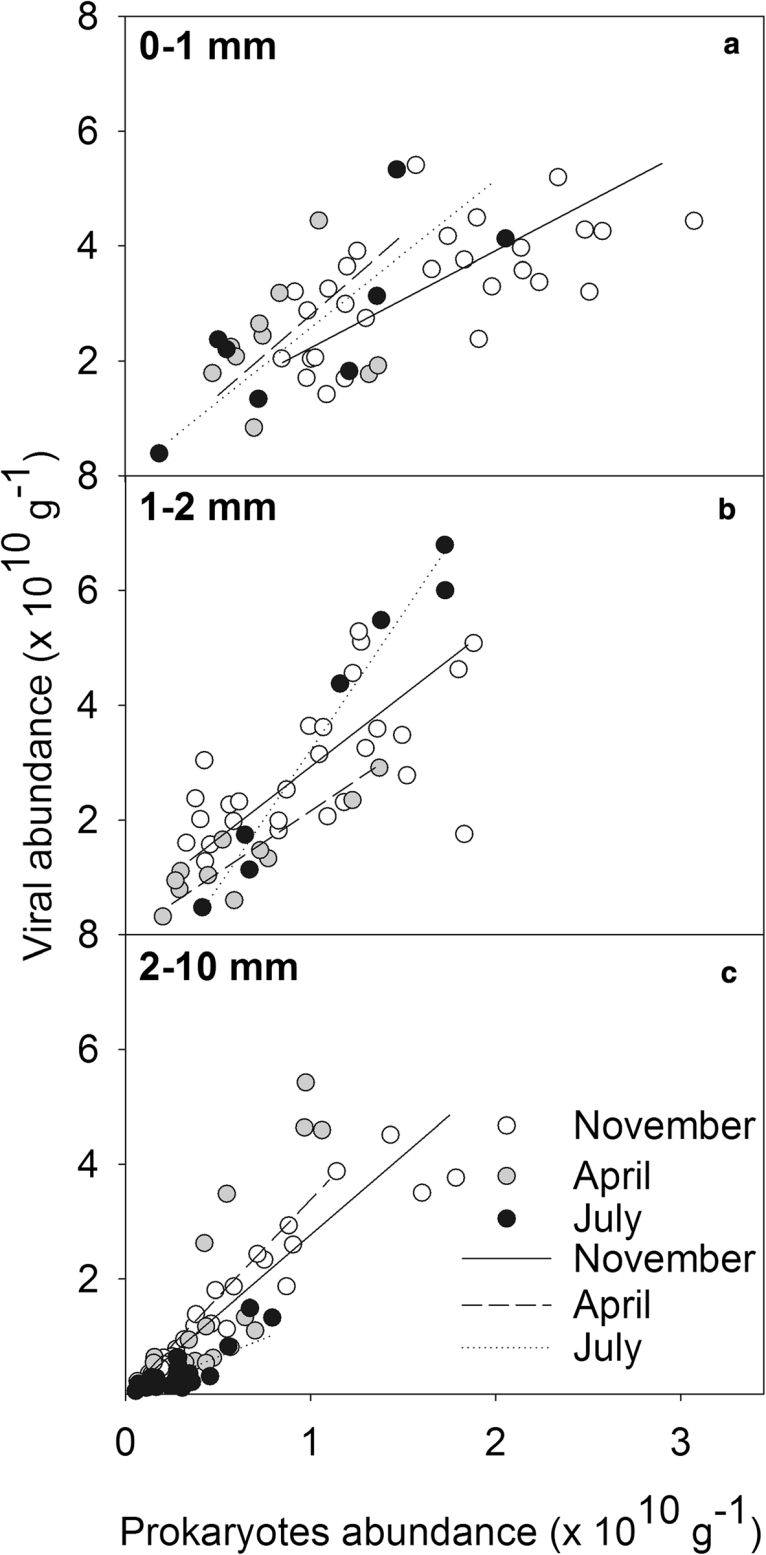
Linear regressions between the abundances of prokaryotes and viruses in three depth zones and per sampling month: **a** 0–1 mm, **b** 1–2 mm, and **c** 2–10 mm

Viral abundances in the top 0–1 mm layer were significantly different from the abundances in the layers just below (1–3 mm), and the abundances in the 1–2 mm layer were significantly different from all layers below 5 mm (Table 1). Prokaryotic abundance was significantly different between the top 0–1 mm and all layers below 2 mm, while the layer 1–2 mm was significantly different from all layers below 4 mm (Table 1). Overall most differences were found between the top 2 or 3 mm and the layers below (2–10 mm) (Table 1). Therefore, in order to identify more detailed spatial patterns in the relationship between the distribution of prokaryotes and viruses, we divided the depth profiles into three depth intervals: 0–1 mm, 1–2 mm and 2–10 mm, representing the top and bottom of the photosynthetic microbial mat, and the sediment under the photosynthetic microbial mat (Fig. 8). Viral abundance showed significant positive linear correlations with prokaryotes abundance for all depth intervals and dates sampled (except in the 0–1 mm layer in April; Table 2). The slopes of regression differed only slightly between the different layers and months. Correlations between viral and prokaryotic abundances were generally weaker in the top photoautotrophic layer (Fig. 8). Neither bacterial nor viral abundances correlated with oxygenic photoautotrophs autofluorescence.

**Table 1:** Statistical analysis of viral and prokaryotic abundances for each depth

Prokaryotes\Viruses										
Depth (mm)	*0.5*	*1.5*	*2.5*	*3.5*	*4.5*	*5.5*	*6.5*	*7.5*	*8.5*	*9.5*
0.5		*< 0.001*	*< 0.05*	*n.s.*	*n.s.*	*n.s.*	*n.s.*	*n.s.*	*n.s.*	*n.s.*
1.5	n.s.		*n.s.*	*n.s.*	*n.s.*	*< 0.05*	*< 0.05*	*< 0.001*	*< 0.001*	*< 0.001*
2.5	< 0.05	n.s.		*n.s.*	*n.s.*	*n.s.*	*n.s.*	*< 0.05*	*< 0.05*	*< 0.001*
3.5	< 0.001	n.s.	n.s.		*n.s.*	*n.s.*	*n.s.*	*n.s.*	*n.s.*	*< 0.05*
4.5	< 0.001	< 0.05	n.s.	n.s.		*n.s.*	*n.s.*	*n.s.*	*n.s.*	*n.s.*
5.5	< 0.001	< 0.05	n.s.	n.s.	n.s.		*n.s.*	*n.s.*	*n.s.*	*n.s.*
6.5	< 0.001	< 0.001	< 0.05	n.s.	n.s.	n.s.		*n.s.*	*n.s.*	*n.s.*
7.5	< 0.001	< 0.001	< 0.001	n.s.	n.s.	n.s.	n.s.		*n.s.*	*n.s.*
8.5	< 0.001	< 0.001	< 0.001	< 0.05	n.s.	n.s.	n.s.	n.s.		*n.s.*
9.5	< 0.001	< 0.001	< 0.001	< 0.05	n.s.	n.s.	n.s.	n.s.	n.s.	

Numbers in italic are the *P*-values for viral abundances, others are the *P*-values for prokaryotic abundances. n.s. stands for not significant. N.s. stands for not significant

**Table 2:** Linear regression analysis information (R^2^, equation, and significance) between viral and prokaryotic abundances in a photosynthetic microbial mat in Schiermonnikoog (as shown in Fig. 8), for the different sampling months (November, April, and July) and depths (0–1, 1–2, and 2–10 mm)

Month	Depth (mm)	R^2^	Equation	P
November	0–1	0.40	Virus = 1.7 Prok + 1.6 × 10^10^	< 0.001
	1–2	0.38	Virus = 2.5 Prok + 1.4 × 10^10^	< 0.001
	2–10	0.90	Virus = 2.8 Prok + 0.1 × 10^10^	< 0.0001
April	0–1	0.02	Virus = 2.8 Prok + 1.9 × 10^10^	n.s.
	1–2	0.81	Virus = 2.2 Prok + 0.2 × 10^10^	< 0.001
	2–10	0.70	Virus = 3.4 Prok − 1.0 × 10^10^	< 0.0001
July	0–1	0.61	Virus = 2.6 Prok + 0.6 × 10^10^	< 0.05
	1–2	0.97	Virus = 4.8 Prok − 1.5 × 10^10^	< 0.0001
	2–10	0.75	Virus = 1.3 Prok − 0.1 × 10^10^	< 0.0001

N.s. stands for not significant

## Discussion

Intertidal photosynthetic microbial mats are dynamic laminated heterogeneous systems with a photoautotrophic layer typically composed of cyanobacteria and diatoms (Dijkman et al. 2010). These mats are characterised by high primary production, high biomass concentration, and high rates of remineralisation and nutrient cycling (De Wit et al. 1989; Van Gemerden 1993; Jonkers et al. 1998). Temperature and light have been shown to influence their productivity, but equally important for the mat development is the reduced impact of grazers (Fenchel 1998).

In an environment with reduced grazing, viruses may potentially play a key role in microbial mortality. In addition, as drivers of nutrient recycling, viruses may support mat regeneration and productivity. Our study shows that the intertidal photosynthetic microbial mats in Schiermonnikoog have high viral abundances, reaching densities of 0.05 to 5.4 × 10^10^ viruses g^−1^. The observed abundances of viruses in the mats are more than 10-fold higher those typically recorded in marine sediments (Danovaro et al. 2008a). Such high viral abundances have only been reported for eutrophic estuarine sediments with high microbial activity (Hewson et al. 2001; Helton et al. 2012). An earlier study of abundances in lagoon microbial mat also showed high abundances (~15 × 10^10^ g^−1^ dry weight) (Pacton et al. 2014). In this study the data was presented as dry weight specific densities (Pacton et al. 2014). Converting the wet weight specific densities obtained in our study to the corresponding densities per gram dry weight using an average water content (42 and 26 % corresponding to 0–1 and 1–2 mm layers, respectively; this study), showed equally high viral (0.2–20 × 10^10^ g^−1^) and prokaryotes (0.2–7.3 × 10^10^ g^−1^) abundances in the top 0–2 mm of the Schiermonnikoog mats.

The high viral abundances found in the present study are possibly the result of the high biological activity found in microbial mats (De Wit et al. 1989; Van Gemerden 1993), as reflected by the high densities of both prokaryotes and oxygenic photoautotrophs in the mats (although inputs from other sources such as sand deposition or rain cannot be excluded).

The higher prokaryotic abundances in the first 0–1 mm of the mat during the November 3D-experiment were most likely linked to the oxygenic photoautotrophic dominance in this top layer. As the viral abundance did not show much variation between the top layer and the 1–2 mm below, our data indicate that prokaryotes were more actively growing in the top layer, or that viral decay was higher in the top than in the 1–2 mm layer below. Prokaryotic and viral activity in coastal marine sediments are generally positively correlated with organic carbon loading (as indicated from carbon mineralization rates), resulting in gradients of increasing viral and bacterial production along trophic gradients (Middelboe and Glud 2006; Middelboe et al. 2006). Moreover, microbial metabolic activity has been found to be highest in the top of the photosynthetic microbial mats and indeed directly linked to organic matter load (Jonkers et al. 2003). Much of the organic matter load that fuels bacterial production is excreted by photosynthetic microbes as exopolymeric substances (Decho et al. 2005). Higher growth rates of prokaryotes have been shown to result in higher viral burst sizes (Middelboe and Glud 2006). However, we found that the VPR in the upper 1 mm layer was mostly lower than in the layers below. Since the surface layer receives higher UV doses and higher radical concentrations due to O_2_ oversaturation (non published data), it seems reasonable to assume that viral decay is higher in the upper layer than the lower layers. This may explain the low average VPR found in the photosynthetic microbial mat as compared to other marine sediment environments (Danovaro et al. 2008a).

The large spatial heterogeneity in oxygenic photoautotrophs distribution and viral and prokaryotic abundances emphasizes that the microbial mats are dynamic structures. This is, to our knowledge, the first study showing distinct spatial separation of cyanobacterial and diatom populations at the microscale. Until now, only patchy spatial dispersal of all oxygenic photoautotrophs combined (inferred from chl *a*) had been observed for the intertidal sediment surface (Seuront and Leterme 2006).

The distribution of prokaryotes and viruses in the mat was also heterogeneous at the microscale, both horizontally and vertically. Such high heterogeneity was previously demonstrated in studies in the water column, biofilms, and sediments (Neu and Lawrence 1997; Seymour et al. 2006; Stewart and Franklin 2008; Carreira et al. 2013). While, energy availability is among the most studied driving forces for spatial heterogeneity in microbial communities in sediments (Blackburn and Fenchel 1999; Fenchel and Glud 2000), other factors such as competition and losses (grazing, viral lysis) may also contribute significantly to spatial heterogeneity. In addition, a recent study has shown that viral activity contributes to the microscale distribution of prokaryotes in intertidal sediments (Carreira et al. 2013). In microbial mats, where grazing is greatly reduced (Fenchel 1998), viruses could constitute the main mortality agent, and consequently hypothesize, that viruses are an important factor driving spatial heterogeneity in prokaryotes and oxygenic photoautotroph distribution patterns in a microbial mats. To test this hypothesis, future studies should focus on measuring viral lysis rates for the different hosts.

In November, at the end of the growth season, the photosynthetic microbial mat was fully developed, with a thick cyanobacterial sheet at the surface and high abundances of prokaryotes and viruses below. As the seasons progressed from November to July, the oxygenic photoautotrophic layer initially decreased with depth (April), but by July it started to increase again. The relatively low microbial abundances at the top in combination with the thin photosynthetic layer in April suggest limited activity of the mat after the winter period, followed by increased activity during summer (July) leading to higher abundances of viruses and prokaryotes in the top layer in July. The high prokaryote and viral abundances observed deeper in the heterotrophic layers in April reflect most likely inter annual variation or ongoing microbial decomposition of mat layers from previous years. We hypothesize that the temporal changes in distribution of viruses and prokaryotes are tightly linked to the photosynthetic activity and extension of the top productive layer of the mat. The general vertical stratification observed agrees with other published works from the same (Bauersachs et al. 2011) or similar areas (Stal et al. 1985; Mir et al. 1991) with different levels of development, due to seasons. This emphasizes the close spatial coupling between autotrophic and heterotrophic processes in these mats.

In general, viruses could be associated with both prokaryotes and oxygenic photoautotrophs in the top 2 mm, as the correlation between viruses and prokaryotes in these layers is weaker than when oxygenic photoautotrophs are absent (2–10 mm), independently of sampling period. However, as photoautotrophic microbial mats contain many different prokaryotes and viruses, future studies should be complemented with more detailed analyses of community composition. This could be accomplished for example, by integrating other methodologies, such as electron microscopy to verify viral morphologies and frequency of infected cells, and metagenomics to verify the microbial diversity. Particularly in such a densely packed, biological and metabolic distinctly diverse ecosystem, molecular techniques are expected to advance our understanding of microbial and viral diversity and host-virus interactions. However, obtaining actual viral mediated mortality rates for this small-scale laminated and spatial heterogeneous mat system will be challenging.

## Conclusion

In summary, oxygenic photoautotrophs, prokaryotes and viruses were distributed in microscale (mm-scale) patches both horizontally and vertically in the photosynthetic microbial mat. The abundance of both prokaryotes and viruses increased in the growing season, in accordance with the greater depth of the photosynthetic layer. The very high viral abundances in the microbial mat, the variation in depth profiles at different times of the year, together with the spatial 3D heterogeneity, suggest that viruses are likely active components in these productive environments. An active role of viruses can be expected to have implications for the development and productivity of the mat and the biogeochemical fluxes within the mat. Our results emphasize the importance for further studies on the role of viruses as regulators of community dynamics and productivity in photosynthetic microbial mats.

## Material and methods

### Site description and sampling

The sampling site is situated in the sandy north-western beach of the coastal island Schiermonnikoog (53° 29' 24.29"N, 6° 8' 18.02"E) in the Wadden Sea (The Netherlands). Intertidal photosynthetic microbial mats are found here due to the dry and windy conditions, with occasional flooding. The microbial mats can be found year-round, but storm, flood and ice cover may destroy the mats in winter (Bauersachs et al. 2011). Samples were collected in November 2012, April and July 2013, over a spatial range of 200 m. Each time, ten samples of 15 × 8 × 4 cm (L × W × H) were collected, placed individually in clean plastic boxes, and transported back to the laboratory, where they were kept at *in situ* conditions until sub-sampling the next day. Three cores of 7 mm diameter were sampled and used for characterisation of water content, organic matter content and particle size. The cores were sliced every 1 mm down to a depth of 10 mm. At each sampling date, four similar 7 mm diameter cores were taken and sub-sampled for depth profiles of prokaryotes and viruses. Additionally, in November 2012 viruses and prokaryotes were also sampled using the 7 mm diameter cores, in a horizontal grid to obtain the 3-dimensional (3D) distribution. For this 3D-distribution subsamples were taken at 20 mm intervals, both in length and width, at two depths (0–1 and 1–2 mm), sampling a total volume of 29400 mm^3^ (210 × 70 × 2 mm).

### Sediment characteristics

Water content of the sediment was determined as the weight loss after drying the sediment at 105 °C for 12 h. Organic matter content was measured as the weight loss of dried samples after combustion at 540 °C for 4 h. Particle-size analyses were performed on the C_org_-free sediment fraction from the 10 slices from 3 cores. Prior to analysis, 20 mL of demineralised water was added to the samples, which were subsequently treated with 5 mL H_2_O_2_ solution (35 %) and boiled until the reaction stopped and the H_2_O_2_ had disintegrated into H_2_O and O_2._ Particle-size analysis was carried out using the Micro-Liquid Module of a Beckmann Coulter Laser Particle Sizer LS13320 at NIOZ, which resulted in 92 particle-size distributions classes from 0.4 to 2000 μm.

### Chlorophyll quantification

Chlorophyll *a* (chl *a*) autofluorescence was recorded with a camera to map oxygenic photoautotrophic biomass distribution and to distinguish between the two major groups of photosynthetic microorganisms: cyanobacteria and diatoms (Bolhuis et al. 2013). Although autofluorescence always occurs via chl *a*, different wavelengths can be used to excite the accessory pigments, thus allowing discrimination of different algal groups based on the excitation light applied. Blue light was used to excite chl *a* and fucoxanthin (~450 nm) found in diatoms (Van den Hoek et al. 1995; Jeffrey et al. 2005). Blue light excitation does not allow the distinction between diatoms and green microalgae, but diatoms tend to dominate over green microalgae in this type of microbial mats (Jonkers et al. 2003; Bolhuis et al. 2013). Moreover, green microalgae were not observed using light microscopy. Amber light was used to excite phycocyanin (590–640 nm) which is exclusively found in cyanobacteria (Van den Hoek et al. 1995).

A cooled CCD 16 bits camera (Tucsen Imaging Technology Co. LTD, China) (1360 × 1024), with a long pass filter (>685 nm) placed in front of the camera was used to photograph the microbial mats during exposure to blue (470 nm) and amber (600 nm) excitation light (Carreira et al. 2015a). Autofluorescence images of blue and amber (Blue-amber ratio, BAR; log scale), were used as an indicator of cyanobacteria dominance (<0), or diatoms dominance (>0). Images were analysed with ImageJ 1.47 m. Autofluorescence values were extracted from the same area as the core sampling for viruses and prokaryotes.

### Viral and prokaryotes abundances

For enumeration of prokaryotes and viruses, 100 mg subsamples were taken from every sampling depth and placed in sterile 2 mL Eppendorf tubes. The subsamples were fixed with 2 % glutaraldehyde final concentration (25 % EM-grade, Merck, diluted in sterile seawater), for 15 min at 4 °C. As storage before extraction results in decreased prokaryotes cell and virus count (Carreira et al. 2015b), subsamples were immediately treated and placed on a filter, after which they were stored. Extraction of prokaryotes and viruses from the sediment samples was performed according to the protocol by Carreira et al. (2015b). In short, subsamples were incubated with 0.1 mM EDTA (final concentration) on ice and in the dark for 15 min, ultrasonicated (Soniprep 150; 50 Hz, 4 μm amplitude, exponential probe) for three cycles of 10 s with 10 s intervals in ice-water. One microliter of each subsample and 1 μL of Benzonase Endonuclease from *Serratia marcescens* (Sigma-Aldrich; > 250 U μL^−1^) were added to 1 mL of sterile MilliQ water (18.2 MΩ), and incubated at 37 °C in the dark for 30 min. The digestion of free nucleic acids was stopped by placing the sample on ice, directly followed by filtration onto a 0.02 μm pore size filter (Anodisc 25, Whatman) and stained according to Noble and Fuhrman (1998) using SYBR Gold (Molecular Probes®, Invitrogen Inc., Life Technologies™, NY, USA). Each filter was rinsed three times with sterile MilliQ and mounted on a glass slide with an anti-fade solution containing 50 % glycerol, 50 % phosphate buffered solution (PBS, 0.05 M Na_2_HPO_4_, 0.85 % NaCl, pH 7.5) and 1 % *p*-phenylenediamine (Sigma-Aldrich) and stored at −20 °C. Viruses and prokaryotes were counted using a Zeiss Axiophot microscope equipped for epifluorescence (100 W mercury lamp, excitation with a BP 450–490, emission LP 515, beam splitter FT 510). Magnification of the ocular was × 12.5 and the oil lens (Plan-NEOFLUAR) was x100. At least 10 fields and 400 viruses and prokaryotes were counted per sample. Abundances were presented per gram of wet weight (as samples were too small, i.e. 100 mg, to conduct simultaneous measurements of abundance and water content). Counting error was 1.5 and 10 % for viruses and prokaryotes respectively.

### Statistical analyses

Linear regression analyses (model II) were performed to obtain the best-fitting coefficients between pairs of variables (Sokal and Rohlf 1995). To determine differences in viral and prokaryotes abundances between depths, ANOVA I analysis with post hoc Tukey HSD tests were performed. Prior to statistical analysis, normality was checked and the confidence level was set at 95 %. All statistical analyses were conducted using SigmaPlot 12.0.

## References

[CR1] Azam F, Malfatti F (2007). Microbial structuring of marine ecosystems. Nat Rev Microbiol.

[CR2] Bauersachs T, Compaoré J, Severin I, Hopmans EC, Schouten S, Stal LJ, Sinninghe Damsté JS (2011). Diazotrophic microbial community of coastal microbial mats of the southern North Sea. Geobiology.

[CR3] Blackburn N, Fenchel T (1999). Influence of bacteria, diffusion and shear on micro-scale nutrient patches, and implications for bacterial chemotaxis. Mar Ecol Prog Ser.

[CR4] Bolhuis H, Fillinger L, Stal LJ (2013). Coastal microbial mat diversity along a natural salinity gradient. PLoS One.

[CR5] Bolhuis H, Cretoiu MS, Stal LJ (2015). Molecular ecology of microbial mats. FEMS Microbiol Ecol.

[CR6] Canfield DE, Thamdrup B, Kristensen E (2005). Aquatic Geomicrobiology.

[CR7] Carreira C, Larsen M, Glud RN, Brussaard CPD, Middelboe M (2013). Heterogeneous distribution of prokaryotes and viruses at the microscale in a tidal sediment. Aquat Microb Ecol.

[CR8] Carreira C, Staal M, Middelboe M, Brussaard CPD (2015). Autofluorescence imaging system to discriminate and quantify the distribution of benthic cyanobacterial and diatom.

[CR9] Carreira C, Staal M, Middelboe M, Brussaard CPD (2015). Counting viruses and bacteria in photosynthetic microbial mats. Appl Environ Microbiol.

[CR10] Castenholz RW, Stal LJ, Caumette P (1994). Microbial mat research: the recent past and new perspectives. Proceedings of the NATO advanced research workshop on structure, development and environment significance of microbial mats, Book G35.

[CR11] Danovaro R, Corinaldesi C, Filippini M, Fisher UR, Gessner MO, Jacquet S, Magagnini M, Velimirov B (2008). Viriobenthos in freshwater and marine sediments: a review. Freshwat Biol.

[CR12] Danovaro R, Dell’Anno A, Corinaldesi C, Magagnini M, Noble R, Tamburini C, Weinbauer M (2008). Major viral impact on the functioning of benthic deep-sea ecosystems. Nature.

[CR13] De Brouwer JFC, Ruddy GK, Jones TER, Stal LJ (2002). Sorption of EPS to sediment particles and the effect on the rheology of sediment slurries. Biogeo Chem.

[CR14] De Wit R, Jonkers HM, Van De Ende FP, Van Gemerden H (1989). *In situ* fluctuations of oxygen and sulphide in marine microbial sediment ecosystems. Neth J Sea Res.

[CR15] Decho AW, Visscherb PT, Reid RP (2005). Production and cycling of natural microbial exopolymers (EPS) within a marine stromatolite. Palaeogeogr Palaeoclimatol Palaeoecol.

[CR16] Des Marais DJ (2003). Biogeochemistry of hypersaline microbial mats illustrates the dynamics of modern microbial ecosystems and the early evolution of the biosphere. Biol Bull.

[CR17] Dijkman NA, Boschker HTS, Stal LJ, Kromkamp JC (2010). Composition and heterogeneity of the microbial community in a coastal microbial mat as revealed by the analysis of pigments and phospholipid-derived fatty acids. J Sea Res.

[CR18] Fenchel T (1998). Formation of laminated cyanobacterial mats in the absence of benthic fauna. Aquat Microb Ecol.

[CR19] Fenchel T, Glud RN (2000). Benthic primary production and O_2_-CO_2_ dynamics in a shallow-water sediment: spatial and temporal heterogeneity. Ophelia.

[CR20] Fenchel T, Kühl M (2000). Artificial cyanobacterial mats: growth, structure, and vertical zonation patterns. Microb Ecol.

[CR21] Fenchel T, King GM, Blackburn TH (2012). Bacterial biogeochemistry: the ecophysiology of mineral cycling.

[CR22] Helton RR, Wang K, Kan J, Powell DH, Wommack KE (2012). Interannual dynamics of viriobenthos abundance and morphological diversity in Chesapeake Bay sediments. FEMS Microbiol Ecol.

[CR23] Hewson I, O’Neil JM, Heil CA, Bratbak G, Dennison WC (2001). Effects of concentrated viral communities on photosynthesis and community composition of co-occurring benthic microalgae and phytoplankton. Aquat Microb Ecol.

[CR24] Jeffrey SW, Mantoura RFC, Bjørnland T, Jeffrey SW, Mantoura RFC, Wright SW (2005). Data for the identification of 47 key phytoplankton pigments. Phytoplankton pigments in oceanography: guidelines to modern methods, Book 10.

[CR25] Jonkers HM, Koopmans GF, Van Gemerden H (1998). Dynamics of dimethyl sulfide in a marine microbial mat. Microb Ecol.

[CR26] Jonkers HM, Ludwig R, De Wit R, Pringault O, Muyzer G, Niemann H, Finke N, De Beer D (2003). Structural and functional analysis of a microbial mat ecosystem from a unique permanent hypersaline inland lake: “La Salada de Chiprana” (NE Spain). FEMS Microbiol Ecol.

[CR27] Jørgensen BB, Revsbech NP, Cohen Y (1983). Photosynthesis and structure of benthic microbial mats: microelectrode and SEM studies of four cyanobacterial communities. Limnol Oceanogr.

[CR28] Middelboe M, Glud RN (2006). Viral activity along a trophic gradient in continental margin sediments off central Chile. Mar Biol Res.

[CR29] Middelboe M, Glud RN, Wenzhöfer F, Oguri K, Kitazato H (2006). Spatial distribution and activity of viruses in the deep-sea sediments of Sagami Bay, Japan. Deep Sea Res (I Oceanogr Res Pap).

[CR30] Mir J, Martinez-Alonso M, Esteve I, Guerrero R (1991). Vertical stratification and microbial assemblage of a microbial mat in the Ebro Delta (Spain). FEMS Microbiol Ecol.

[CR31] Neu TR, Lawrence JR (1997). Development and structure of microbial biofilms in river water studied by confocal laser scanning microscopy. FEMS Microbiol Ecol.

[CR32] Noble RT, Fuhrman JA (1998). Use of SYBR Green I for rapid epifluorescence counts of marine viruses and bacteria. Aquat Microb Ecol.

[CR33] Pacton M, Wacey D, Corinaldesi C, Tangherlini M, Kilburn MR, Gorin GE, Danovaro R, Vasconcelos C (2014) Viruses as new agents of organomineralization in the geological record. Nat Commun 5 10.1038/ncomms5298 24989676

[CR34] Paerl HW, Pinckney JL (1996). A mini-review of microbial consortia: their roles in aquatic production and biogeochemical cycling. Microb Ecol Health Dis.

[CR35] Seuront L, Leterme C, Kromkamp JC, De Brouwer JFC, Blanchard GF, Forster RM, Créach V (2006). Microscale patchiness in microphytobenthos distributions: evidence for a critical state. Functioning of microphytobenthos in estuaries.

[CR36] Seymour JR, Seuront L, Doubell M, Waters RL, Mitchell JG (2006). Microscale patchiness of virioplankton. J Mar Biol Assoc UK.

[CR37] Siem-Jørgensen M, Glud RN, Middelboe M (2008). Viral dynamics in a coastal sediment: seasonal pattern, controlling factors and relations to the pelagic-benthic coupling. Mar Biol Res.

[CR38] Sokal RR, Rohlf FJ (1995). Biometry the principles and practice of statistics in biological research, Vol.

[CR39] Stal LJ (1995). Physiological ecology of cyanobacteria in microbial mats and other communities. New Phytol.

[CR40] Stal LJ, Van Gemerden H, Krumbein WE (1985). Structure and development of a benthic marine microbial mat. FEMS Microbiol Ecol.

[CR41] Stewart PS, Franklin MJ (2008). Physiological heterogeneity in biofilms. Nat Rev Microbiol.

[CR42] Van den Hoek C, Mann DG, Jahns HM (1995). Algae, An introduction to phycology.

[CR43] Van Gemerden H (1993). Microbial mats: a joint venture. Mar Geol.

[CR44] Ward DM, Bateson MM, Ferris MJ, Kühl M, Wieland A, Koeppel A, Cohan FM (2006). Cyanobacterial ecotypes in the microbial mat community of Mushroom Spring (Yellowstone National Park, Wyoming) as species-like units linking microbial community composition, structure and function. Philos Trans R Soc Lond Ser B: Biol Sci.

